# Changes in attachment dimensions during the treatment of acute post-traumatic stress disorder in sexually assaulted Brazilian women

**DOI:** 10.3389/fpsyg.2023.1325622

**Published:** 2023-12-07

**Authors:** Mariana Rangel Maciel, Vinicius Fernando Calsavara, Cecilia Zylberstajn, Marcelo Feijo Mello, Bruno Messina Coimbra, Andrea Feijo Mello

**Affiliations:** ^1^Program for Research and Care on Violence and PTSD (PROVE), Department of Psychiatry, Universidade Federal de São Paulo (UNIFESP), São Paulo, Brazil; ^2^Samuel Oschin Comprehensive Cancer Institute, Cedars-Sinai Medical Center, Los Angeles, CA, United States; ^3^Department of Psychiatry, Amsterdam UMC, University of Amsterdam, Amsterdam Public Health Research Institute and Amsterdam Neuroscience Research Institute, Amsterdam, Netherlands; ^4^Faculty of Social and Behavioural Sciences, Department of Methodology and Statistics, Utrecht University, Utrecht, Netherlands

**Keywords:** post-traumatic stress disorder, attachment, interpersonal psychotherapy, treatment, sexual assault

## Abstract

**Introduction:**

Attachment patterns are established during early childhood; however, extreme experiences throughout life may change this structure, either toward attachment security or insecurity. We analyzed changes in attachment dimensions in women with acute post-traumatic stress disorder (PTSD) following sexual assault, that were randomized to a 14-week treatment with either the medication sertraline or Interpersonal Psychotherapy.

**Methods:**

Seventy-four adult women who presented significant reduction in PTSD symptoms across the trial responded to the Revised Adult Attachment Scale at baseline, on week 8 of treatment, and at the end of the trial, on week 14. We fitted a generalized linear model to explain the attachment anxiety and avoidance scores at baseline. A generalized linear mixed model investigated how attachment dimensions changed over time. Socioeconomic data, treatment type, history of childhood trauma, and PTSD severity over the 14-week period were the considered covariates.

**Results:**

At baseline, attachment anxiety was associated with a history of early trauma. Attachment anxiety remained stable during the follow-up. Attachment avoidance, on the other hand, significantly increased from baseline to week 14. Higher avoidance was observed in patients with higher total PTSD scores and on the cluster of hyperarousal symptoms. Races other than White (black, mixed-race, or Asian) and younger age were associated with higher attachment avoidance.

**Discussion:**

Contrary to our expectations, attachment avoidance increased during follow-up, indicating changes in the interpersonal realm beyond the symptoms of PTSD.

## 1 Introduction

Sexual assault is frequent and distributed globally (Abrahams et al., 2014; World Health Organization, 2021). In Brazil, recent data show a consistent increase in rape reports over the past 10 years, from 44.000 in 2011 to 75.000 in 2022. Nonetheless, estimates indicate that only 8.5% of incidents are reported, suggesting actual numbers may reach approximately 800,000 cases annually (Cerqueira et al., 2021; Bueno et al., 2023).

There are multiple forms of sexual violence; sexual assault refers to any sexual contact or behavior without explicit consent. Rape is defined as sexual assault with penetration. Sexual assault victims are at a high risk of developing clinical and psychiatric disorders, such as post-traumatic stress disorder (PTSD), depression, substance abuse, and anxiety disorders (Elliott et al., 2004; Dworkin et al., 2017; Lake et al., 2022; Stea et al., 2023). The prevalence of PTSD after sexual assault is approximately 50%, and this type of trauma is also associated with poorer PTSD outcomes, as the disease tends to be more severe and chronic (Steenkamp et al., 2012; Elklit and Christiansen, 2013; Möller et al., 2014).

PTSD is a condition that may develop after a life-threatening experience. The traumatic event is required to consider the diagnosis; symptoms include intrusive memories of the experience, avoidance of stimuli that remind them of the event, social isolation, anhedonia, hyperarousal, irritability, sleep disturbances (Yehuda et al., 2015). Most individuals that go through life-threatening experiences do not develop PTSD. Pre-trauma characteristics, specificities of the traumatic event itself, and post-trauma factors may increase or decrease the magnitude of risk (Brewin et al., 2000). Among the pre-trauma characteristics under consideration is attachment style (Clark and Owens, 2012; Bruno et al., 2019; Marshall and Frazier, 2019).

Attachment style is one of the most extensively studied characteristics that shape how humans respond to stress. When John Bowlby developed the theory, which was later strengthened by the field research done by Mary Ainsworth and colleagues, attachment was conceptualized as a behavioral system based on early experiences with caregivers, which determined a matrix called “internal working model” – a template of how comfortable that individual would be in intimate relations, and how positive his or her views and expectations of themselves and others would predominantly be (Bowlby, 1973; Bretherton, 1992).

Observational studies in children demonstrated that attachment patterns could be described by two linear functions, anxiety and avoidance; the same functions were used to determine types of attachment behavior in adults, expanding research to other life stages (Ainsworth et al., 1978; Hazan and Shaver, 1987). Attachment anxiety refers to the degree to which a person is worried about the availability of others in times of need, resulting in hyperactivation of the attachment system; attachment avoidance refers to the difficulty in closeness and relying on others for support, leading to deactivating strategies. Securely attached individuals display low anxiety and low avoidance. The anxiety dimension can be conceptualized as a model of self, while the avoidance dimension represents as a model of others. Individuals characterized by secure attachment have a positive model of both self and others, whereas individuals with insecure attachment may have a negative model of self (anxiety), others (avoidance), or both (anxious avoidant) (Griffin and Bartholomew, 1994).

The attachment pattern established in childhood launches the individual into a developmental pathway, that tends to stability but is subject to change depending on cumulative history along the way (Sroufe and Waters, 1977). As a result, both continuity and discontinuity of attachment style across the lifespan are possible, as indicated by longitudinal studies (Sroufe, 2005; Van Assche et al., 2013; Chopik et al., 2019). The initial matrix serves as a prototype that can be later modified to a certain point, depending on experiences that follow during adult life (Pinquart et al., 2013). Severe interpersonal violence, socioeconomic disadvantage, and chronic disease can potentially increase attachment insecurity (Waters et al., 2000; Bachem et al., 2019). Evidence indicates that samples more exposed to adversities may display more frequent changes in the structure, likely due to the instability within their life circumstances and relationships (Weinfield et al., 2000, 2004).

Conversely, positive life changes and relationships and social support may help change attachment styles toward security (Travis et al., 2001; Pace et al., 2019; Slade and Holmes, 2019). Furthermore, it has been replicated that psychotherapy may enhance attachment security (Lahav and Elklit, 2016; Diamond et al., 2023) as research findings have reported attachment style changes in time-limited treatments with several psychotherapy orientations (Muller and Rosenkranz, 2009; Kinley and Reyno, 2013). The literature on psychotherapeutic treatment for PTSD offers relevant data, as improving social bonds, interpersonal relationships, and attachment are possible mechanisms through which psychotherapy may exert its effects (Ravitz et al., 2008; Coyne et al., 2018).

Secure attachment has consistently shown a link to resilience, while insecure attachment styles are identified as risk factors for developing psychopathology (Fonagy et al., 1996; Darling Rasmussen et al., 2019). In the face of stress and adversity, securely attached individuals tend to maintain emotional regulation, effectively seek social support, activate internalized representations of attachment figures, and balance emotions and cognitions in their behavior (Ensink et al., 2016). In contrast, insecurely attached individuals often experience difficulties in self-regulation, exhibit unstable relationships, display more negative affectivity, and suffer from higher levels of global distress – all risk factors for psychopathology (Mikulincer and Shaver, 2012).

The relationship between attachment and post-traumatic reactions, particularly PSTD, has been explored as a risk factor for the development of pathological reactions, alone or interacting with biological and psychological characteristics (Lim et al., 2012; Tamman et al., 2021; Jittayuthd and Karl, 2022). Attachment has been shown to interfere with the response and adherence to treatment (Forbes et al., 2010). Overall, studies indicate that insecure attachment styles are associated with a higher risk of developing PTSD and a worse prognosis of the disorder (Levy et al., 2011; Woodhouse et al., 2015; Ferrajão, 2022). Considering that the relationship of attachment and trauma is bidirectional, it is also relevant to investigate the effect of trauma and PTSD on attachment styles. This has been explored longitudinally in cohorts of veterans with PTSD (Franz et al., 2014; Mikulincer et al., 2014). Taken together, the evidence suggests that insecure attachment may function as both a risk factor for and be influenced by PTSD (Castro-Vale et al., 2020). However, there is limited evidence investigating longitudinal changes in attachment styles in patients undergoing treatment for PTSD.

In light of the documented association between PTSD, its treatment, and attachment within existing literature, our study aimed to longitudinally explore attachment dimensions over a 14-week period of post-traumatic stress disorder intervention. The research encompassed a cohort of 74 women who recently experienced sexual assault (rape or attempted rape) and received treatment using either sertraline or Interpersonal Psychotherapy adapted for PTSD (IPT-PTSD; Markowitz, 2016). We hypothesized that attachment anxiety and avoidance would be greater in patients with higher levels of childhood and adolescent trauma, with greater PTSD severity, younger age, and lower income.

Subsequently, we measured whether attachment fluctuated during follow-up and which covariates might be related to possible attachment changes. We hypothesized that attachment dimensions would shift toward security; that is, reduced avoidance and anxiety dimensions scores. Considering that IPT works on interpersonal relations as a means to improve PTSD symptoms (Lipsitz and Markowitz, 2013), we hypothesized that changes in attachment would be more prominent in the psychotherapy group. Psychiatric treatment (consultations and the use of medication sertraline) does not target attachment directly but may affect it indirectly through PTSD improvement. Other variables included in the model were: PTSD symptoms, age, history of early trauma, socioeconomic status, racial background, and marital status. These characteristics have been shown to have a possible impact on attachment styles (Ehrlich et al., 2022; Kazmierski et al., 2023).

## 2 Materials and methods

### 2.1 Participants

Participants were referred to us by a women’s health hospital in São Paulo. Women admitted to this hospital with a history of sexual assault (defined as forced penetration, attempted forced penetration, or alcohol/drug-facilitated forced or attempted penetration) that had occurred one to six months prior to admission were requested to fill in the National Stressful Events Survey Short Scale for PTSD (LeBeau et al., 2014). If this screening measure was positive, they were referred to our outpatient clinic. A telephone call from our team assessed initial eligibility criteria; an appointment with psychologists or psychiatrists from the research team was then scheduled, for a full clinical evaluation and subsequent tests. From 149 women referred to our service, 34 refused to participate in the study, 38 were not eligible, and 3 could not be located. A total of 74 women diagnosed with PTSD after sexual assault were enrolled in the trial between January 2016 and April 2019. The complete study protocol has been described by Coimbra et al. (2020).

The inclusion criteria were as follows: (1) age 18–45 years; (2) sexual assault experienced 1–6 months before evaluation; (3) current PTSD diagnosis on the Mini-International Neuropsychiatric Interview (MINI) (Sheehan et al., 1998; Amorim, 2000); (4) CAPS-5 score > 26, indicating a current PTSD diagnosis; and (5) Ethics Review Board-approved signed informed consent. Exclusion criteria were as follows: (1) psychiatric or psychotherapeutic treatment for the present disorder; (2) severe suicidal risk, evaluated in a clinical interview by a trained researcher; (3) pregnancy; (4) chronic corticosteroid use; (5) unstable medical condition or neurologic disease; (6) Inability to understand the informed consent or research protocol; (7) substance use disorder within the previous six months; and (8) bipolar disorder or schizophrenia. This study was approved by the Research Ethics Committee.

### 2.2 Measures

The initial interview included (a) a sociodemographic questionnaire developed for the study, including age, race, relationship status, income, living conditions, and educational level; (b) Clinician-Administered Post-Traumatic Stress Disorder Scale-5 (Weathers et al., 2018); (c) Childhood Trauma Questionnaire (Bernstein et al., 1994); and (d) Revised Adult Attachment Scale – Close Relationships version (Collins and Read, 1990; Collins, 1996). The study data were stored in REDCap, a secure web-based research application.

The RAAS is a self-reported measure of adult attachment conceptually derived from Hazan and Shaver’s prototype for adult attachment (Hazan and Shaver, 1987). According to the authors, it can be scored on three (close, dependent, and anxious) or two (avoidance and anxiety) dimensions. We chose to use the latter, facilitating comparisons with other studies. There are several self-report measures of attachment, and many yield results in the form of these two dimensions (Mikulincer and Shaver, 2016). Continuous values were used for this analysis, resulting in scores ranging from 1 to 5 for each dimension. If a categorical viewpoint was used, scores above 3 would be considered positive for each dimension. Cronbach’s Alpha for anxiety, close and dependent subscales are, respectively, 0.72, 0.69 and 0.75; test–retest reliability indexes are 0.52, 0.68 and 0.72 (Collins and Read, 1990). The instrument has been validated for the Brazilian population (Teixeira et al., 2019).

PTSD symptoms were quantified using the CAPS-5. The scale generates a total score that can be summed to indicate the overall severity of the disorder; the threshold for establishing a PTSD diagnosis was 26. It comprises 20 symptom-related questions divided into four groups, one for each diagnostic cluster: five items for intrusion symptoms, two items for avoidance, seven items for alterations in cognition and mood, and six items for hyperarousal. Each symptom is quantified from 0 (absent) to 4 (extreme/incapacitating), and a severity rating of 2 or higher implies that the criteria are considered present. The CAPS-5 has demonstrated high internal consistency (α = 0.88), and high interrater and test–retest reliability coefficients (κ = 0.78 and 0.83, respectively); the correlation with other valid measures of PTSD symptoms is strong, indicating high construct validity (Weathers et al., 2018). The Portuguese version of the scale has been validated in Brazil (Oliveira-Watanabe et al., 2021).

The CTQ is a widely used retrospective, self-report measure of childhood maltreatment. Four factors are identified: physical and emotional abuse, physical neglect, emotional neglect, and sexual abuse. The instrument presents 28 items, and response to each item is rated on a 5-point Likert-type scale. Results can be analyzed for each trauma type or as a total score – a continuous total score was the measure that entered our analysis. Cronbach’s Alpha for each factor range from 0.78 to 0.94, and 0.95 for the total scale, showing high internal consistency, and test–retest reliability coefficients range from 0.80 to 0.88 (Bernstein et al., 1994). The instrument shows high convergence to interview measures of early trauma and has been translated to Portuguese (Grassi-Oliveira et al., 2006).

### 2.3 Procedures

After inclusion, the patients were randomized to receive either IPT-PTSD (*n* = 38) or pharmacological treatment with sertraline (*n* = 36). The duration of the randomized clinical trial was 14 weeks. IPT was delivered in 14 weekly 50-min sessions following the IPT-PTSD manual (Markowitz, 2016). The sertraline dose ranged from 50 to 200 mg daily and was titrated according to the clinician’s understanding of the patient’s needs and tolerance. Appointments were scheduled at baseline and weeks 2, 4, 8, and 14. Pharmacotherapy sessions lasted 15–30 min and focused on targeting PTSD symptoms and exploring medication benefits and side effects. Both sertraline and IPT-PTSD patients could receive low-dose quetiapine (25–50 mg), risperidone (0.5–2.0 mg), or zolpidem CR (12.5 mg) for insomnia or PTSD-related psychotic symptoms.

An evaluator blinded to the treatment condition assessed patients at baseline and weeks 8 and 14. Independent appointments were scheduled for these assessments, that were performed by a psychologist who was otherwise not involved in the clinical trial. The standardized instruments used at the initial visit were repeated at weeks 8 and 14, except for the sociodemographic questionnaire and the CTQ, which were only applied at the initial visit.

The results of this clinical trial have been published and are beyond the scope of this study (Proença et al., 2022). We assessed the PTSD symptoms and attachment dimensions of the entire sample at baseline and at weeks 8 and 14 to evaluate the eventual attachment changes across treatments.

### 2.4 Statistical analysis

We described baseline patient characteristics as absolute and relative frequencies for qualitative variables and as the mean, standard deviation, median, and minimum and maximum for quantitative variables. Bivariate associations between two qualitative variables were assessed using Fisher’s exact test or the chi-squared test – qualitative variables used in the model were race, marital status and income (classified in two categories). Quantitative variables were compared between the two groups using the t-test if the normality assumption was satisfied; otherwise, the Mann–Whitney U test was applied. The Shapiro-Francia test was applied to evaluate the normality assumption.

Univariable and multivariable generalized linear models (GLMs) were fitted considering: (i) avoidance and (ii) anxiety dimensions as outcomes, and age, income, CTQ score, race, treatment group (SER or IPT-PTSD), relationship status, and CAPS-5 total score at baseline as covariates. We also fitted multivariable generalized linear mixed models (GLMMs) considering the attachment dimensions of anxiety and avoidance, measured by the RAAS over time, as outcomes, and the same covariates previously mentioned. In the mixed models, the CAPS-5 score was considered as a time-dependent covariate: scores from the baseline, from week 8 and week 14 were added in the model. The subjects’ random effects describing the repeated measures were considered. The presence of an interaction term between time and patient characteristics was tested using a likelihood ratio test. Sensitivity analyses were conducted by employing each of the four CAPS-5 subscales—symptoms of intrusion, avoidance, alterations in cognition and mood, and hyperarousal—as time-dependent covariates instead of using the total CAPS-5 score collected over time. The gamma distribution with identity or log-link functions was utilized to identify the factors associated with the avoidance score, whereas the normal distribution with the identity link function was used to model the anxiety score as a function of the covariates in the GLMs and GLMMs.

We chose to use valid cases in all analyses and refrained from applying imputation strategies to handle missing data. Profile analysis showed great variability in individual patterns, rendering the imputation perilous.

All hypothesis tests were two-sided with a 5% significance level. Thus, results with *p*-values lower than 0.05 were considered statistically significant. All statistical analyses were performed using R version 4.1 (R Core Team, 2020).

## 3 Results

The baseline characteristics of the treatment groups are shown in Table 1. No statistically significant differences were observed between the treatment groups at baseline. Race was reported by the patients to be white (*n* = 32), black/mixed-race (*n* = 41), and Asian (*n* = 1). Because of the small number of patients in each distinct category, we grouped the categories Black/mixed-race and Asian, and two categories were considered for the statistical analysis.

**Table 1:** Demographic and psychometric characteristics in treatment groups.

Variable
Category or summary measure	SER (*n* = 36)	IPT (*n* = 38)
Age (year)	Mean (SD)	23.99 (6.20)	25.28 (7.21)
	Median (Min-Max)	21.43 (18.02–44.42)	22.99 (18.04–42.33)
Marital Status	Single	24 (66.7%)	26 (68.5%)	Divorced/ separated	1 (2.8%)	1 (2.6%)
	Married/ partnership	11 (30.5%)	11 (28.9%)
	Race	Black/mixed-race	24 (66.7%)	17 (44.7%)
White		12 (33.3%)	20 (52.7%)
Asian		0 (0%)	1 (2.6%)
Annual income (USD)	Mean (SD)	2178.11 (2855.8)	2935.11 (2960.6)
	Median (Min-Max)	1485.7 (314.3–14857.1)	2102.9 (0–11428.6)
CTQ total score	Mean (SD)	42.61 (15.43)	40.51 (16.6)
	Median (Min-Max)	37.5 (25–91)	34 (25–96)
CAPS-5 total score baseline	Mean (SD)	43.58 (8.33)	41.55 (9.87)
	Median (Min-Max)	43.5 (26–60)	40.50 (22–61)
CAPS-5 total score week 8	Mean (SD)	30.38 (15.14)	34.17 (14.45)
	Median (Min-Max)	35.5 (20.5–41)	36.5 (20.5–46)
CAPS-5 total score week 14	Mean (SD)	25.5 (14.48)	31.43 (16.18)
	Median (Min-Max)	23.5 (15.5–37)	32 (13–45)
RAAS anxiety baseline	Mean (SD)	2.84 (1.34)	2.44 (1.02)
	Median (Min-Max)	2.67 (1–5)	2.5 (1–5)
RAAS avoidance baseline	Mean (SD)	3.16 (0.77)	2.82 (0.83)
	Median (Min-Max)	3.17 (1.67–4.83)	2.92 (1.08–4.42)
RAAS anxiety week 8	Mean (SD)	2.72 (1.16)	2.97 (1.0)
	Median (Min-Max)	2.83 (1–4.33)	2.83 (1.33–4.5)
RAAS avoidance week 8	Mean (SD)	3.65 (0.72)	3.54 (0.66)
	Median (Min-Max)	3.79 (2.25–4.5)	3.67 (2.25–4.58)
RAAS anxiety week 14	Mean (SD)	2.19 (1.09)	2.65 (1.17)
	Median (Min-Max)	2 (1–4.5)	2.67 (1–4.5)
RAAS avoidance week 14	Mean (SD)	3.2 (0.66)	3.23 (0.52)
	Median (Min-Max)	3.29 (1.83–4.33)	3.33 (1.75–4.08)

SER, sertraline; IPT, interpersonal psychotherapy; RAAS, revised adult attachment scale; SD, standard deviation; CTQ, childhood trauma questionnaire; CAPS-5, clinician-administered PTSD scale.

Sexual assault was classified as rape (with penetration) in 88% of women, and in 94.6% of the cases, the aggressor was unknown to the victim. The prevalence of drug-facilitated sexual assault was 27%. Early abuse and neglect were also extensive in our sample, as indicated by the total CTQ scores in Table 1.

Of the 74 women enrolled, 48 (64.9%) completed the intermediate assessment (week 8) and 46 (62.2%) completed the clinical trial (week 14), generating attrition rates of 37.8% from baseline to week 14. The research team excluded six women, for reasons such as pregnancy, elevated suicidal risk, or initiating concomitant treatment elsewhere. Twenty-two women dropped out of study (29.7%); 15 of them did so very early in the follow-up, attending only the first or second visit. There were no statistically significant differences between completers and dropouts regarding treatment type, PTSD symptoms, attachment dimensions scores, and other baseline characteristics except for age – women who dropped out of the study were younger, with mean age 22.27 (4.60) vs. 26.10 (7.50), (*p* < 0.05). The RAAS was evaluated in 73 women at baseline, 25 (34.2%) at week 8, and in 45 (61.6%) women who completed the trial – one of the completers missed the evaluation visit at week 14.

The only significant association in the linear models was attachment anxiety (RAAS anxiety) and a history of early trauma (CTQ total score; *p* < 0.05). No significant associations were observed with attachment avoidance. The results of the fitted models are presented in Supplementary Material (Part 1).

Two multivariable mixed models were fitted to evaluate the changes in attachment dimensions across the 14 weeks of follow-up. Tables 2, 3 present the results of the multivariable generalized linear mixed models for the outcomes of attachment avoidance and anxiety, respectively. The interaction terms between time and (i) CAPS-5 scores, (ii) treatment arm, and (iii) race were tested, and no statistically significant interactions were found. Attachment anxiety did not show statistically significant variations across time and none of the examined covariates had an impact on the results. Attachment avoidance, on the other hand, increased over time (*p* < 0.0001); the increase was greater for patients with higher CAPS-5 scores, younger patients, and those who reported a race other than white (Black, mixed-race, or Asian).

**Table 2:** Multivariable linear mixed model for avoidance.

Variable	Category	Estimate (*β*)	SE	DF	*t* value	Value of *p*
Intercept		2.299	0.412	58.715	5.575	<0.0001
Treatment arm	SER	Ref				
	IPT	−0.219	0.133	44.459	−1.644	0.107
Timepoint	Baseline	Ref				
	Week 8	0.724	0.160	93.670	4.528	<0.0001
	Week 14	0.595	0.150	105.278	3.978	0.0001
CAPS-5	1-unit	0.024	0.005	99.462	4.368	<0.0001
CTQ total score	1-unit	0.003	0.005	52.172	0.539	0.592
Age (years)	1-unit	−0.025	0.012	45.629	−2.019	0.049
Relationship status	Married/partnership	Ref				
	Divorced/separated	0.352	0.370	29.967	0.952	0.349
	Single	0.071	0.150	47.564	0.471	0.640
Race	White	Ref				
	Other (black/mixed-race, Asian)	0.327	0.147	52.758	2.225	0.030
Annual income (USD)	≤ 2 minimum wages	Ref				
	> 2 minimum wages	0.259	0.213	44.399	1.214	0.231

Df: degrees of freedom; SE: standard error; SER: sertraline; IPT: interpersonal psychotherapy; CAPS-5, clinician-administered PTSD Scale-5. Ref: Reference category.

**Table 3:** Multivariable generalized linear mixed model for anxiety.

Variable	Category	Estimate (*β*)	SE	*t* value	Value of *p*
Intercept		0.818	0.428	1.910	0.056
Treatment arm	SER	Ref			
	IPT	−0.129	0.151	−0.854	0.393
Timepoint	Baseline	Ref			
	Week 8	0.066	0.061	1.083	0.279
	Week 14	−0.039	0.062	−0.628	0.530
CAPS-5	1-unit	0.001	0.003	0.450	0.653
CTQ total score	1-unit	0.005	0.005	0.906	0.365
Age (years)	1-unit	−0.002	0.014	−0.154	0.878
Relationship status	Married/partnership	Ref			
	Divorced/separated	0.203	0.509	0.400	0.690
	Single	−0.020	0.168	−0.119	0.905
Race	White	Ref			
	Other (black/mixed-race, Asian)	−0.078	0.158	−0.493	0.622
Annual income (USD)	≤ 2 minimum wages	Ref			
	> 2 minimum wages	−0.324	0.242	−1.339	0.181

Df: degrees of freedom; SE: standard error; SER: sertraline; IPT: interpersonal psychotherapy; CAPS-5, clinician-administered PTSD scale-5. Ref: Reference category.

We constructed an additional model using the CAPS-5 subscales for each cluster of PTSD symptoms instead of the total score. Other covariates remained unchanged. The only PTSD symptom cluster significantly related to attachment avoidance was hyperarousal (Cluster E). The tables that depict the results of this model are provided in Supplementary Materials, Part 2. Both groups showed a similar improvement in PTSD symptoms – see Proença et al. for the complete description of the results of the clinical trial (Proença et al., 2022).

**Figure 1:**
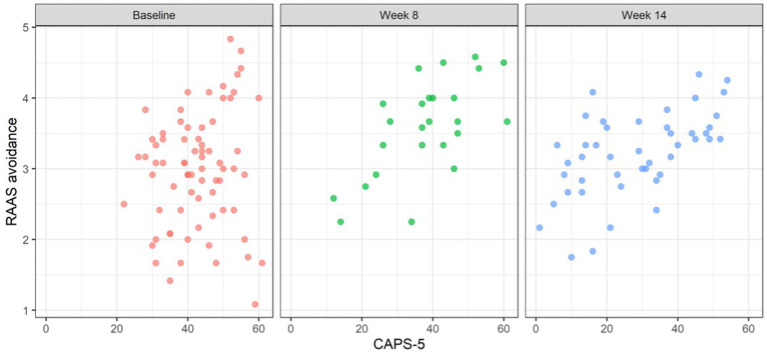
Scatterplot of attachment avoidance and CAPS-5 scores over time.

**Figure 2:**
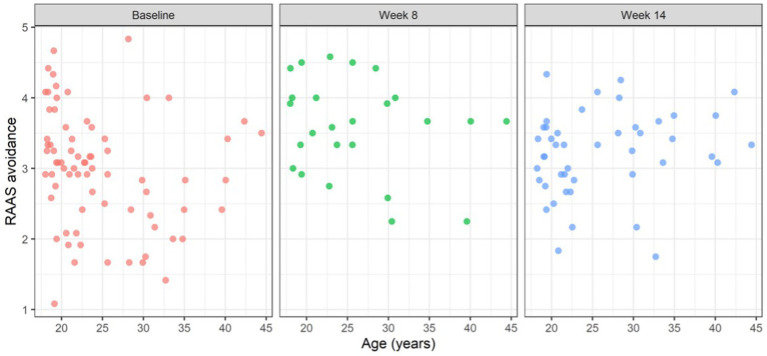
Scatterplot of attachment avoidance and age over time.

**Figure 3:**
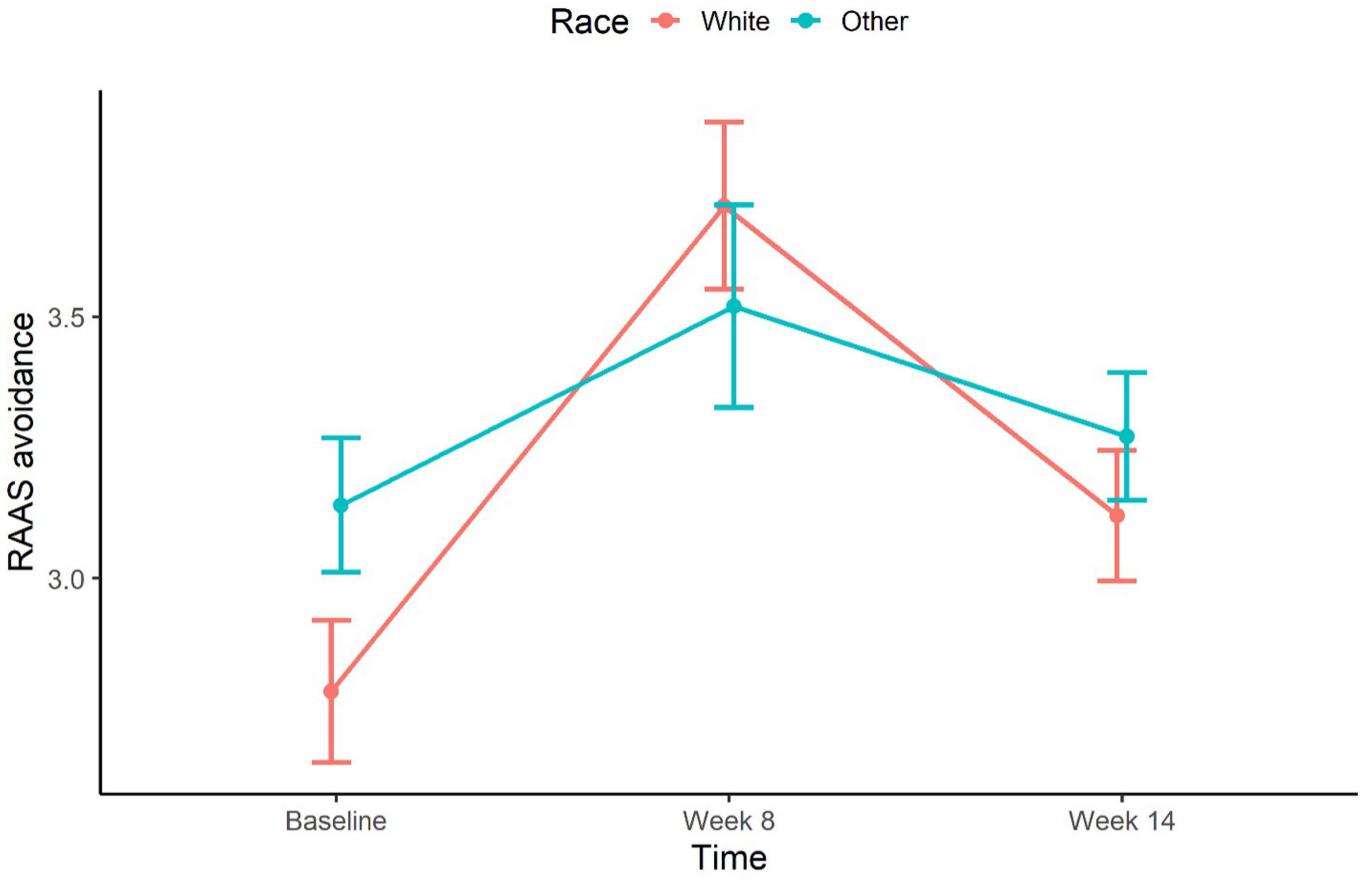
Mean profile of attachment avoidance over time by race. Error bars represent the standard error.

Figures 1–3 illustrate RAAS avoidance and anxiety results across follow-up.

## 4 Discussion

In this study, we examined attachment styles in a sample of adult women with PTSD after a recent sexual assault who were treated for 14 weeks with either IPT or sertraline. Attachment was measured at baseline and at weeks 8 and 14, as well as PTSD symptoms. Contrary to our hypothesis, attachment anxiety remained stable throughout the follow-up period, and attachment avoidance slightly increased equally in both treatment groups. This increased attachment avoidance was associated with higher PTSD severity, races other than white (Black, mixed-race, Asian), and younger age. When the PTSD symptoms were broken down into clusters, the cluster associated with increased attachment avoidance was hyperarousal (cluster E).

We found higher levels of attachment insecurity in our sample than generally reported in epidemiological data; approximately 40% presented with secure attachment at baseline, while population studies find 60% (Mickelson et al., 1997; Bakermans-Kranenburg et al., 2009). However, recent data evaluating college students in the United States indicate a possible rise in insecure attachment to levels similar to those found in our study (Konrath et al., 2014). To the best of our knowledge, there are no data on the Brazilian population. Considering that we evaluated only women who developed PTSD after sexual violence (not resilient), the higher proportion of insecure attachment is possibly a reflection of its role as a risk factor for the development of the disorder (Mikulincer et al., 2015).

The women in our study reported high levels of childhood trauma, which is strongly associated with attachment insecurity (Egeland and Sroufe, 1981). The findings regarding childhood trauma are consistent with epidemiological data from the Brazilian population (Zanoti-Jeronymo et al., 2009; Soares et al., 2016). The only characteristic that significantly explained the attachment dimensions at baseline was early trauma, which was related to attachment anxiety. None of the covariates analyzed explained attachment avoidance.

Contrary to our expectations, we did not find a decrease in attachment anxiety and/or avoidance during the follow-up period, accompanying an improvement in PTSD symptoms. The increase in attachment avoidance in our sample was associated with higher PTSD symptoms across the trial, which may reflect more severely compromised patients who were less responsive to treatment. Younger age and race other than white (black, mixed-race, and Asian) were also associated with higher attachment avoidance. Younger women could experience a greater impact on their beliefs regarding trust and comfort in proximity to others after experiencing severe interpersonal trauma. Regarding race, it has been discussed in the literature that differences in attachment might be mediated by socioeconomic status rather than ethnic or racial background (Bakermans-Kranenburg et al., 2004); in Brazil, however, adversities faced by black/mixed-race population also include higher violence exposure, difficulties in accessing healthcare, and other impacts of structural racism (Instituto Brasileiro de Geografia e Estatística (IBGE), 2019; Oliveira et al., 2020; Cerqueira et al., 2021).

It is worth investigating whether attachment avoidance confers an evolutionary advantage to individuals or groups. Social defense theory posits that, in dangerous settings, the sense of security may increase vulnerability by postponing the recognition or reaction to threats, and insecure attachment strategies – either activating the attachment system, as seen in anxiously attached individuals, or deactivating the system, as seen in avoidant individuals – could increase the chances of survival (Ein-Dor et al., 2010). Violence against women is a major health problem in Brazil and worldwide, and enduring this unsafe environment may require adaptive behavior (Mesman et al., 2016). Further research in the mental health and social sciences must explore these hypotheses.

This study has several limitations. Attrition rates were significant: there were almost 30% of actual dropouts, most of them in the first weeks of treatment. Adherence in PTSD studies is challenging, as the symptoms themselves may hinder the patients’ ability to attend treatment; previous evidence has reported that younger age is associated with higher dropout rates in PTSD studies, which is consistent with our findings (Hale et al., 2019). Our evaluation was limited to women who experienced sexual assault and subsequently developed PTSD, excluding those who did not develop PTSD. Thus, our study focused on individuals following trajectories of either disease and recovery or evolution toward chronicity, rather than examining resilience to trauma. Therefore, we were unable to determine whether the attachment pattern found at baseline was affected by an acute PTSD diagnosis, rather than reflecting long-term characteristics. We assessed attachment through a self-report measure; this type of assessment has been extensively used in the field, particularly because of its feasibility over interview measures, although these methods emphasize different phenomena, and their psychometric properties have been questioned (Maunder and Hunter, 2009; Ravitz et al., 2010; Justo-Núñez et al., 2022). It is also possible that the self-reported measures at all time points were affected by the experience of severe interpersonal trauma. Longitudinal studies with representative samples of the Brazilian population may be more suitable for investigating attachment patterns and trajectories across the lifespan.

Another limitation of our study lies in the relatively narrow timeframe, potentially limiting our ability to detect attachment changes. The treatment targeted improvements in PTSD symptoms and not changes in attachment, particularly in the sertraline group, where patients received medication and psychiatric treatment as usual. Nevertheless, short-term interventions in psychotherapy studies, such as CBT for panic disorder (Zalaznik et al., 2019; Lange et al., 2021) and IPT for depression in adolescents and adults (Bernecker et al., 2014; Gunlicks-Stoessel et al., 2017) have shown alterations in attachment. A pilot study of 29 veterans treated for PTSD with IPT observed improvement in separation anxiety symptoms, which can be considered a proxy for insecure attachment (Milrod et al., 2020). Despite our focus on interpersonal relations and social support within the IPT-PTSD treatment group, we could not observe changes in the dimensions of attachment style structure.

One of the biggest strengths of this study is the homogeneity of our sample, particularly considering the trauma type – few PTSD studies with civilian populations have investigated a single type of violence. Research on attachment and PTSD in developing countries is scarce, and this is to our knowledge the first study investigating this association in Brazil. Exploring attachment, rather than limiting it to diagnostic criteria, expands our understanding of the psychological functioning in traumatized individuals. Considering the high rates of urban violence and childhood adversities, further exploration of attachment patterns in the Brazilian population may be crucial for tailoring specific interventions for violence-related disorders.

We assessed anxiety and avoidance dimensions representing attachment styles in adult women undergoing a 14-week PTSD treatment following recent sexual assault. Both IPT-PTSD and sertraline effectively reduced PTSD symptoms; however, attachment avoidance levels increased over time while attachment anxiety remained stable. Our results indicate that difficulties in the interpersonal realm after severe interpersonal trauma should be investigated and addressed independently from the diagnosis, as they may pose a heavy and lasting burden on these women.

## Data availability statement

The original contributions presented in the study are included in the article/Supplementary material, further inquiries can be directed to the corresponding author.

## Ethics statement

The studies involving humans were approved by the Universidade Federal de São Paulo Ethics Comitee. The studies were conducted in accordance with the local legislation and institutional requirements. The participants provided their written informed consent to participate in this study.

## Author contributions

MRM: Conceptualization, Data curation, Formal analysis, Investigation, Methodology, Writing – original draft, Writing – review & editing. VC: Formal analysis, Methodology, Writing – review & editing. CZ: Data curation, Writing – review & editing. MFM: Conceptualization, Funding acquisition, Project administration, Resources, Supervision, Visualization, Writing – review & editing. BC: Conceptualization, Formal analysis, Project administration, Supervision, Visualization, Writing – review & editing. AM: Conceptualization, Formal analysis, Funding acquisition, Investigation, Resources, Supervision, Visualization, Writing – review & editing.
