# Supplementary material for: Changes in attachment dimensions during the treatment of acute post-traumatic stress disorder in sexually assaulted Brazilian women
Source: Front Psychol. 2023 Dec 7;14:1325622. doi: 10.3389/fpsyg.2023.1325622 (PMC10734689; doi:10.3389/fpsyg.2023.1325622)
Supplement: Supplementary file 1 [file Data_Sheet_1.pdf]

Supplementary Material

Part 1

Table 4: Multivariable generalized linear model for attachment anxiety (RAAS-anxiety)

Variable	Estimate (β)	95% CI	p-value
**Treatment arm**			
**SER**	Ref		
**IPT**	-0.195	-0.804;0.383	0.507
**Age (years)**	-0.002	-0.047;0.053	0.946
**Annual Income (USD)**			
**<= 2 Minimum wages**	Ref		
**>2 Minimum wages**	-0.614	-1.320;0.181	0.133
**Race**			
**White**	Ref		
**Black, mixed-race, Asian**	-0.193	-0.793;0.404	0.538
**Relationship status**			
**Married/Partnership**	— Ref		
**Divorced/Separated**	0.404	-1.230;3.434	0.695
**Single**	0.193	-0.488;0.828	0.544
**CTQ total score**	0.029	0.001;0.062	0.035
**CAPS-5 total score**	0.016	-0.020;0.052	0.365

SE, Standard Error; SER, sertraline; IPT, interpersonal psychotherapy; CAPS-5, Clinician-Administered PTSD Scale-5. A gamma distribution with an identity link function was considered in the modeling.

Table 5: Multivariable linear regression model for attachment avoidance (RAAS-avoidance)

Variable	Estimate (β)	95% CI	p-value
**Treatment arm**			
**SER**	Ref		
**IPT**	-0.294	-0.698-0.109	0.158
**Age**	-0.037	-0.075-0.002	0.065
**Annual Income**			
**≤ 2 Minimum wages**	Ref		
**>2 Minimum wages**	0.235	-0.422-0.892	0.486
**Race**			
**White**	Ref		
**Black, mixed-race, Asian**	0.355	-0.071-0.781	0.108
**Relationship status**			
**Married/Partnership**	Ref		

**Divorced/Separated**	0.526	-0.761-1.813	0.427
**Single**	0.221	-0.230-0.671	0.341
**CTQ total score**	-0.001	-0.015-0.013	0.854
**CAPS-5 total score**	0.014	-0.010-0.038	0.249

SE, Standard Error; SER, sertraline; IPT: Interpersonal Psychotherapy; CTQ: Childhood Trauma Questionnaire; CAPS-5: Clinician-Administered PTSD Scale-5; CI = Confidence Interva

Part 2

Table 6: Multivariable linear mixed model for attachment avoidance (CAPS scores divided into symptom clusters)

Variable	Estimate (β)	SE	t value	p-value
**Intercept**	2.220	0.419	5.301	<0.0001
**Treatment arm**				
**SER**	Ref			
**IPT**	-0.213	0.131	-1.630	0.111
**Timepoint**				
**Baseline**	Ref			
**Week 8**	0.743	0.179	4.154	0.0001
**Week 14**	0.606	0.174	3.475	0.0007
**Re-experiencing**	0.023	0.025	0.920	0.360
**Avoidance**	-0.015	0.053	-0.291	0.771
**Negative Mood**	0.005	0.016	0.334	0.739
**Hyperarousal**	0.071	0.023	3.049	0.003
**CTQ total score**	0.002	0.005	0.494	0.624
**Age (years)**	-0.025	0.012	-2.069	0.045
**Relationship status**				
**Married/Partnership**	Ref			
**Divorced/Separated**	0.467	0.373	1.253	0.221
**Single**	0.125	0.152	0.825	0.414

**Race**				
**White**	Ref			
**Black, mixed-race, Asian**	0.370	0.146	2.537	0.015
**Annual income (USD)**				
**≤ 2 minimum wages**	Ref			
**> 2 minimum wages**	0.247	0.209	1.184	0.244

SE: Standard Error; SER: sertraline; IPT: Interpersonal Psychotherapy; CTQ: Childhood Trauma Questionnaire

Table 7: Multivariable linear mixed model for attachment anxiety (CAPS scores divided into symptom clusters)

Variable	Estimate (β)	SE	t value	p-value
**Intercept**	0.738	0.441	1.673	0.094
**Treatment arm**				
**SER**	Ref			
**IPT**	-0.125	0.156	-0.802	0.422
**Timepoint**				
**Baseline**	Baseline	Ref		
**Week 8**	Week 8	0.073	0.071	1.030
**Week 14**	Week 14	-0.034	0.078	-0.435
**Re-experiencing**	0.005	0.011	0.399	0.690
**Avoidance**	0.022	0.023	0.976	0.329
**Negative Mood**	-0.015	0.008	-1.920	0.055
**Hyperarousal**	0.014	0.010	1.329	0.184
**CTQ total score**	0.005	0.005	0.944	0.345
**Age (years)**	-0.003	0.015	-0.197	0.844
**Relationship status**				
**Married/Partnership**	Ref			
**Divorced/Separated**	0.267	0.529	0.504	0.614
**Single**	0.022	0.174	0.127	0.899

**Race**				
**White**	Ref			
**Black, mixed-race, Asian**	-0.060	0.162	-0.372	0.710
**Annual income (USD)**				
**≤ 2 minimum wages**	Ref			
**> 2 minimum wages**	-0.333	0.249	-1.337	0.181

SE: Standard Error; SER: sertraline; IPT: Interpersonal Psychotherapy; CTQ: Childhood Trauma Questionnaire
